# Withdrawal of infliximab therapy in ankylosing spondylitis in persistent clinical remission, results from the REMINEA study

**DOI:** 10.1186/s13075-019-1873-3

**Published:** 2019-04-05

**Authors:** Mireia Moreno, Jordi Gratacós, Vicenç Torrente-Segarra, Raimon Sanmarti, Rosa Morlà, Caridad Pontes, Maria Llop, Xavier Juanola, REMINEA study Group, Arturo Rodriguez-de-la-Serna, Victoria Hernandez, Elena Riera, Vera Ortiz, Teresa Clavaguera, Patricia Reyner, Miquel Sala, Agustí Sellas, Concepció Pitarch, Delia Reina, Jordi Blanco, Montse Centelles, Ramon Figuls, Mario Gelman, Xavier Arasa, Maria Bonet, Manel Ciria

**Affiliations:** 1 Rheumatology Department, Parc Taulí Hospital Universitari, I3PT, Universitat Autònoma de Barcelona, 08208 Sabadell, Barcelona Spain; 2 Rheumatology Department, Hospital Comarcal de l’Alt Penedès, Vilafranca, Barcelona Spain; 3 Rheumatology Department, Hospital Clínic de Barcelona, Universitat de Barcelona, Barcelona, Spain; 4 Rheumatology Department, Hospital del Vendrell, Tarragona, Spain; 5 Clinical Pharmacology Department, ParcTaulí Hospital Universitari, I3PT, Universitat Autònoma de Barcelona, Sabadell, Barcelona Spain; 6 Rheumatology Department, Hospital Universitari de Bellvitge, IDIBELL, Universitat de Barcelona, L’Hospitalet de Llobregat, Barcelona Spain

**Keywords:** Ankylosing spondylitis, Anti-TNF therapy, Disease activity, Treatment withdrawal

## Abstract

**Background:**

Recent data suggest that anti-TNF doses can be reduced in ankylosing spondylitis (AS) patients. Some authors even propose withdrawing treatment in patients in clinical remission; however, at present there is no evidence to support this.

**Objective:**

To assess how long AS patients with persistent clinical remission remained free of flares after anti-TNF withdrawal and to evaluate the effects of treatment reintroduction. We also analyze the characteristics of patients who did not present clinical relapse.

**Methods:**

Multicenter, prospective, observational study of a cohort of patients with active AS who had received infliximab as a first anti-TNF treatment and who presented persistent remission (more than 6 months). We recorded at baseline and every 6–8 weeks over the 12-month period the age, gender, disease duration, peripheral arthritis or enthesitis, HLA-B27 status, BASDAI, CRP, ESR, BASFI, and three visual analogue scales, spine global pain, spinal night time pain, and patient’s global assessment.

**Results:**

Thirty-six out of 107 patients (34%) presented persistent remission and were included in our study. After treatment withdrawal, 21 of these 36 patients (58%) presented clinical relapse during follow-up. Infliximab therapy was reintroduced and only 52% achieved clinical remission, as they had before the discontinuation of infliximab; in an additional 10%, reintroduction of infliximab was ineffective, obliging us to change the anti-TNF therapy. No clinical or biological factors were associated with the occurrence of relapse during the follow-up.

**Conclusions:**

Two thirds of patients in clinical remission presented clinical relapse shortly after infliximab withdrawal. Although the reintroduction of infliximab treatment was safe, half of the patients did not present the same clinical response that they had achieved prior to treatment withdrawal.

## Background

Axial spondyloarthritis (axSpA) comprises a group of chronic, immune-mediated inflammatory diseases characterized by the predominance of inflammation in the sacroiliac joints and spine. Involvement of peripheral skeletal sites and extra-articular manifestations, such as uveitis, psoriasis, or inflammatory bowel disease (IBD), may develop during the course of the disease [1]. Ankylosing spondylitis (AS) is the most representative disease in this group; its burden has recently been recognized as severe, frequently leading to invalidity, work loss, and social impairment [1, 2].

The introduction of biological therapy has undoubtedly been an important step forward in improving the quality of life, activity, functionality, metrology, and most extra-articular manifestations in patients with AS and other forms of spondyloarthropathy [1]. Around 60% of patients treated with an anti-TNF achieve a good clinical response; however, according to the Assessment of Spondyloarthritis International Society (ASAS), only 20–30% achieve criteria of clinical partial remission in which the patient is apparently asymptomatic [3, 4].

Data from uncontrolled studies and from clinical practice support the possibility of reducing anti-TNF doses below the normal levels licensed in AS patients with good clinical response, and especially in patients in clinical remission [5–7]. In this sense, a recent consensus paper from the Spanish Society of Rheumatology and the Spanish Society of Hospital Pharmacy [8] stressed the possibility of withdrawing anti-TNF treatment in some patients who maintain good clinical response after intensive reduction of anti-TNF treatment [8], but the evidence supporting this recommendation is lacking.

In this sense, previous classic studies have suggested the need to maintain anti-TNF therapy indefinitely, as the withdrawal of this treatment is associated with disease reactivation in around 90% of patients within 12 months, independently of the anti-TNF used or the previous duration of treatment [9]. The factors associated with these reactivations include the high clinical activity at the time of treatment suspension, especially when measured through C-reactive protein (CRP), as well as age and disease duration [9]. However, the heterogeneity of the clinical symptoms of the AS patients included the different drugs and doses of anti-TNF prescribed prior to the withdrawal of the treatment and especially the lack of homogeneous criteria of clinical remission in the different studies published all mean that the issue remains highly controversial.

More recently, data from a multicenter, randomized, double-blind study in patients with non-radiographic axSpA, who initially achieved sustained remission, showed that continued therapy with adalimumab was associated with a significant maintenance of remission compared with treatment withdrawal [10].

Since anti-TNF treatment also presents drawbacks, such as its high cost and the possibility of long-term side effects, it seems reasonable to plan a time-limited treatment for some patients. The aim of our study was to analyze the consequences of the clinical decision to withdraw anti-TNF treatment in a very homogeneous group of AS patients. In this study, we included only data from AS patients who received infliximab as a first anti-TNF treatment and who presented persistent clinical remission (at least 6 months) in accordance with our pre-established definition [11]. The focus of the study was to assess how long patients maintained a good clinical response defined as the absence of flare after anti-TNF withdrawal, and to define the characteristics of patients who did not present clinical relapse.

## Methods

We conducted a prospective observational study in 23 hospitals with Rheumatology Services in Catalonia, north-eastern Spain, a region with seven million inhabitants. The study included a cohort of patients with active AS who had received infliximab as a first anti-TNF treatment. The study was authorized by the Ethics Committee of the participating hospitals, and all patients gave informed consent prior to taking part in the study.

Patients were included in the cohort and started infliximab treatment if they were aged 18 or over, had a diagnosis of AS according NY criteria, presented a disease duration longer than 1 year, and fulfilled criteria for anti-TNF treatment according to the Guidelines of the Spanish Society of Rheumatology (SER) (BASDAI ≥ 4, despite treatment with 2 nonsteroidal anti-inflammatory drugs (NSAID) for a minimum of 3 months at full dose) [12]. After the infliximab induction period, all patients who presented persistent clinical remission (BASDAI ≤ 2, normal CRP, and absence of active arthritis and/or enthesitis and/or any other extra-articular manifestation during the last 6 months in the absence of any additional steroid and/or NSAID treatment) were included in a prospective study of infliximab withdrawal. Patients with any other definitive diagnosis of spondyloarthritis (psoriatic arthritis, inflammatory bowel disease, or reactive arthritis) were excluded, as were those with any concomitant rheumatic disease that might modify the clinical evaluation of the disease activity.

During the study, in accordance with standard clinical practice, we prospectively recorded the following variables at baseline, and every 6–8 weeks over a follow-up period of 12 months: age, gender, disease duration, the presence and number of peripheral arthritis or enthesitis, the presence of HLA-B27, the BASDAI (Bath Ankylosing Spondylitis Disease Activity Index), CRP, erythrocyte sedimentation rate (ESR), Bath Ankylosing Spondylitis Functional Index (BASFI), and three visual analogue scales (VAS), spine global pain, spinal night time pain, and patient’s global assessment. In the pre-study phase, as a secondary objective, we also retrospectively recorded the data from all the cohort of patients with active AS who had initiated treatment with infliximab.

Clinical relapse was defined in any time period as newly appearing BASDAI ≥ 4 and/or PCR ≥ 0.8 mg/dl. For patients with relapse after treatment withdrawal, infliximab treatment was reintroduced without an induction phase or any previous premedication. At the end, a final visit was performed in all patients included in the study.

Frequencies and percentages were given for subjects who achieved initial remission after infliximab treatment and for subjects who did not present a clinical flare after treatment withdrawal during follow-up. Other parameters including baseline and clinical data during follow-up were described by frequency and percentage, mean and standard deviation (SD), median and 25 and 75 percentiles, and 95% confidence intervals [95%CI], as appropriate. No inferential analysis was conducted.

## Results

**Fig. 1:**
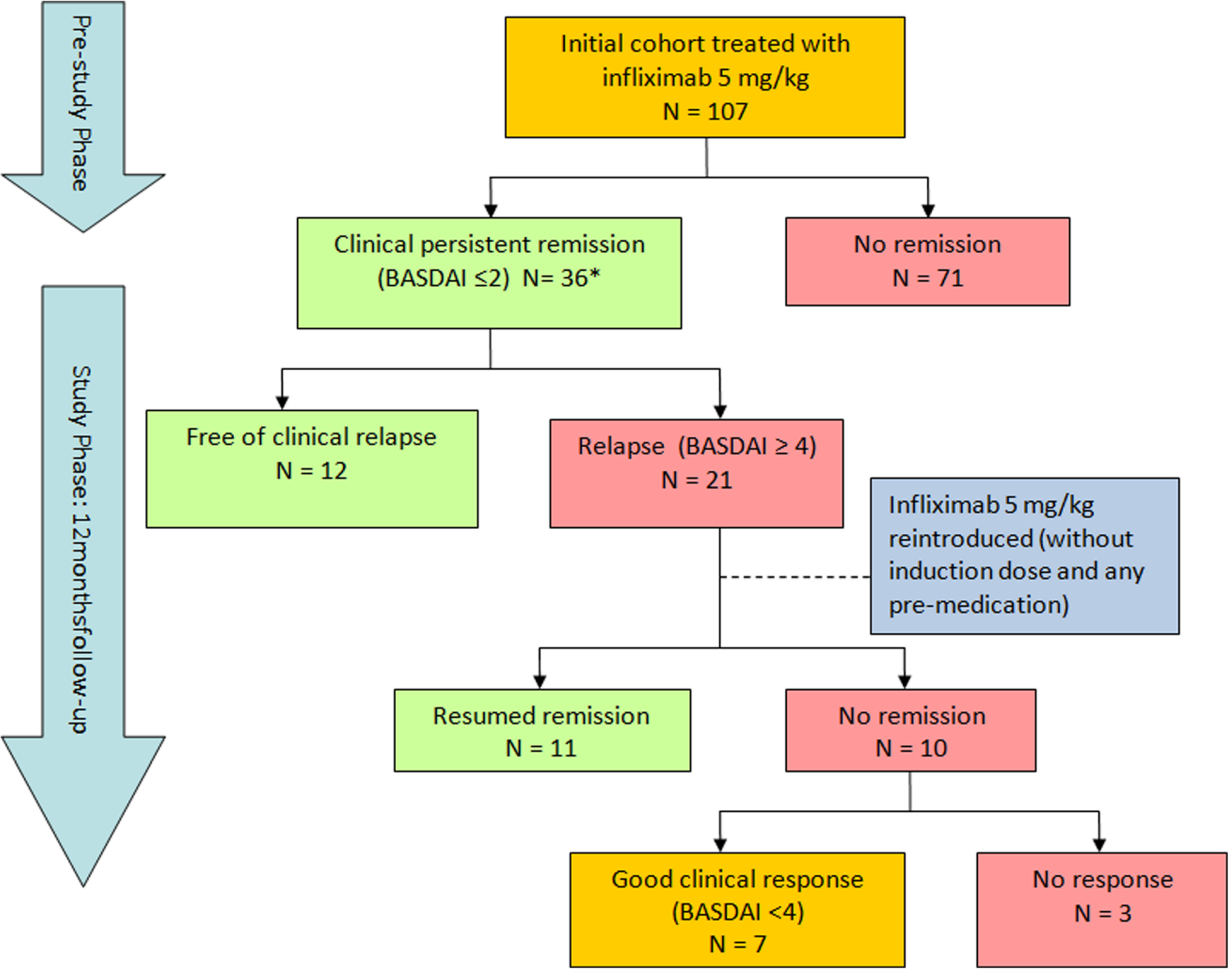
Flow chart of the patient outcomes included in the REMINEA study. Clinical persistent remission: BASDAI ≤ 2 and normal C-protein reactive (CRP) and the absence of active arthritis and/or enthesitis and/or any other extra-articular manifestation during the last 6 months in the absence of any additional steroid and/or NSAID treatment. Relapse: any time period as newly appearing BASDAI ≥ 4 and/or CRP ≥ 0.8 mg/dl. Good clinical response: BASDAI< 4 and/or CRP < 0.8 mg/dl. Asterisk indicates 3 patients in the study phase of 12 months of follow-up were lost to follow-up

One hundred and seven patients, 72% male, with a first infliximab prescription, were retrospectively identified. The main characteristics of these patients are shown in (Table 1). Among these patients, 36 (34%) achieved persistent clinical remission and were included in the prospective study, so infliximab treatment was then discontinued. The period of clinical remission before treatment withdrawal ranged in all cases between 6 and 12 months. After treatment withdrawal, only 12 out of these 36 subjects (33.3%) remained free of clinical relapse during the follow-up. Overall, 21 of these 36 patients (58.3%) presented clinical relapse (three patients were lost during the follow-up study). Half of the relapses appeared within 6 months of infliximab withdrawal. In the 21 patients who presented clinical relapse, infliximab therapy was reintroduced and 11 (52%) again achieved clinical remission, but ten (48%) did not. Of these ten patients, in seven, the reintroduction of infliximab was associated with good clinical response (absence of flare, BASDAI < 4 and/or CRP < 0.8 mg/dl), but in three (14%), the treatment was ineffective, and we had to change to another anti-TNF treatment (Fig. 1). The reintroduction of infliximab was safe, and no important side effects or infusion reactions were recorded.

**Table 1:** Baseline characteristics of the cohort of 107 patients who started infliximab treatment

	Number of valid values	Baseline characteristics
Age (years), mean ± SD	104	41.8 ± 12.0
Disease duration (years)^†^, mean ± SD male sex, *n* (%) HLA-B27 positive, *n* (%)	91,107 77	11.9 ± 10.4 75 (70.1%) 69 (89.6%)
Modified Schober test (cm), mean ± SD	87	3.7 ± 1.9
Fingertip to floor distance (cm), mean ± SD	101	19.6 ± 13.9
Number of swollen joints¸ mean ± SD	107	1.13 ± 2.2
CRP (mg/dl), mean ± SD	103	2.23 ± 2.82
ESR (mm/h), mean ± SD	105	35.3 ± 28.0
VAS nocturnal spinal pain (cm), mean ± SD	99	5.7 ± 2.8
VAS spinal pain(cm), mean ± SD	98	6.3 ± 2.4
VAS patient global (cm), mean ± SD	99	7.2 ± 1.8
BASDAI score (cm), mean ± SD	102	6.2 ± 1.9
BASFI score (cm)¸ mean ± SD	100	4.9 ± 2.7

*n* number, *SD* standard deviation, *CRP* C-reactive protein, *ESR* erythrocyte sedimentation rate, *VAS* patient’s rating of pain by visual analogue scale ranging from 0 (none) to 10 (worst), *BASDAI* Bath Ankylosing Spondylitis Disease Activity Index, *BASFI* Bath Ankylosing Spondylitis Functional Index

^†^50% of patients had less than 10 years of disease duration

Analyzing the retrospective data, we found that age (39 ± 12 vs 43 ± 14 years, *p* = 0.05), disease duration (9 ± 8 vs 14 ± 11 years, *p* = 0.02), and CRP at the start of infliximab treatment (3.41 ± 3.65 vs 1.63 ± 2.10, p = 0.02) were associated, in the pre-study period, with clinical remission under infliximab treatment. However, unfortunately, we did not find any statistical differences between any clinical or biological parameter in patients who remained free of clinical relapse during follow-up after treatment withdrawal compared with those who did not (Table 2).

**Table 2:** Comparison of significant variables between patients on infliximab according to remission status and patients after infliximab withdrawal according to the absence of clinical relapse

	Infliximab treatment			Infliximab withdrawal		
	Clinical persistent remission* (*n* = 36)	No remission (*n* = 71)	*P* value	Clinical relapse** (*n* = 21)	Free of clinical relapse (*n* = 12)	*P* value
Age (years), mean ± SD	38.52 ± 11.96	3.39 ± 11.81	0.05	40 ± 12	37 ± 12	ns
Disease duration (years)†, mean ± SD	8.94 ± 8.09	13.69 ± 11.29	0.02	9 ± 8.0	9 ± 9.0	ns
CRP (mg/dl), mean ± SD	3.41 ± 3.65	1.63 ± 2.10	0.01	3.9 ± 3.5	3.0 ± 3.1	ns
BASDAI score (cm), mean ± SD	5.95 ± 1.85	6.4 ± 1.82	ns	6 ± 2.0	6 ± 2.0	ns
BASFI score (cm)¸ mean ± SD	4.35 ± 2.44	5.18 ± 2.81	ns	4 ± 3.0	5 ± 2.0	ns

*SD* standard deviation, *CRP* C-reactive protein, *BASDAI* Bath Ankylosing Spondylitis Disease Activity Index, *BASFI* Bath Ankylosing Spondylitis Functional Index

*Clinical persistent remission: BASDAI ≤ 2 and normal C-protein reactive (CRP) and the absence of active arthritis and/or enthesitis and/or any otherextra-articular manifestation during the last 6 months in the absence of any additional steroid and/or NSAID treatment, refers to patients under IFX treatment

**Relapse: any time period as newly appearing BASDAI ≥ 4 and/or CRP ≥ 0.8 mg/dl after treatment with IFX withdrawal

^†^50% of patients had less than 10 years of disease duration

## Discussion

This is the first prospective study to show that the majority of longstanding AS patients in persistent clinical remission presented clinical relapse after infliximab withdrawal within the following 12 months. Moreover, although the reintroduction of infliximab was safe and effective in most cases, around half of the patients did not achieve remission after treatment reintroduction and in an additional 10% the treatment was ineffective, obliging us to change the prescription.

Data from clinical practice and registries have suggested that in patients with sustained clinical remission (i.e., more than 6 months), reducing the treatment dose may be a desirable therapeutic goal [5–7]. For example, the recent EULAR guidelines [13] incorporate the tapering of biological therapy for these patients as a new recommendation, even though the data supporting this policy are limited due to the absence of randomized controlled studies. Recently our group have been communicated a randomized pragmatical study demonstrating the no inferiority of a regime of dose reduction compared with full doses in these patients [14].

Many previous studies have suggested that treatment withdrawal in AS patients leads to a reactivation of the disease [8, 15–17]. Nonetheless, in most studies, withdrawal is performed in patients who are not in clinical remission, and some of them even present high CRP serum levels [15, 16]. Recently, a controlled and randomized study in non-radiographic axSpA patients reported in patients who achieved sustained remission with adalimumab more reactivation of the disease in the treatment withdrawal compared with the control arm (patients without suspension of anti-TNF) [10]. However, some official recommendations, based only on clinical practice and in the expert opinion, suggest the possibility of withdrawing treatment in AS patients with persistent clinical remission after a notable reduction in anti-TNF therapy [8, 18].

The data we reported in AS patients were in agreement with the results previously reported by Landewé et al. in non-radiographic axSpA [10]. Unfortunately, in our study as did Landewé et al., the withdrawn of treatment was performed without dose reduction. However, all the patients previous dose reduction were in persistent remission without any analgesic or anti-inflammatory treatment that could mask the clinical symptoms.

Few studies are focused on the clinical response to re-treatment after withdrawal of anti-TNF therapy in patients with axSpA, suggesting as overall that the reintroduction of treatment is comparable to the previously observed [15, 19, 20]. In contrast, our data indicate that the reintroduction of biological therapy (without previous premedication), although it was safe, only half of the patients achieved clinical remission, as they had before the discontinuation of anti-TNF. These data are in agreement with data recently reported by Landewé et al. in non-radiographic axSpA using adalimumab [10]. Furthermore, in our study, in 10% of the patients, the re-treatment was ineffective, obliging us to change the anti-TNF therapy.

The patients we included in the study presented definite AS and had received only infliximab treatment as first-line anti-TNF therapy. The baseline characteristics of our patients are quite similar to those in previous studies of anti-TNF therapy (22, 23). The baseline clinical characteristics we found to be associated with clinical remission after infliximab treatment—younger age, short disease duration, and high CRP levels—were in agreement with the previously published data [21]. Unfortunately, a complete study of clinical and biological factors associated with the presence of relapse during the following 12 months did not yield any positive results. The sample size of our study seems to be the main factor associated with the negative results observed; however, other larger studies also failed to obtain any results in this regard [10, 20].

Overall, the results we reported here suggest that the decision to withdraw treatment should be taken with considerable caution, and it seems unreasonable to propose withdrawal as an objective of the treatment strategy, at least at present, in the absence of any objective predictive factors of persistent clinical remission after treatment withdrawal.

The study has certain limitations that must be mentioned. The sample size is too small to assess factors related to the persistence of remission or the presence of a flare after treatment withdrawal; however, other larger study also failed in this subject [10]. Similarly, since all the patients were in treatment with infliximab, the results need to be corroborated in other anti-TNF agents, but the results published by Landewé et al. [10] using adalimumab are quite similar. Furthermore, our schedule of treatment did not incorporate a strategy of infliximab reduction doses before treatment withdrawal, so we cannot definitively rule out the possibility of withdrawal treatment in patients under persistent remission after intensive doses reduction. The clinical remission period before withdrawal of infliximab treatment (6 to 12 months) does not exclude the possibility of some different results in patients with a longer period of time in clinical remission. Finally, the study began before the publication of the definite new ASAS remission and relapse criteria; however, the criteria applied are widely accepted and used in the clinical practice.

## Conclusion

In summary, this is the first prospective trial performed in a homogeneous cohort of AS patients to evaluate the effect of anti-TNF withdrawal in patients presenting persistent clinical remission. Our data of clinical relapse during the first 12 months in the majority of patients in AS patients are in agreement with the study in non-radiographic axSpA previously published. Moreover, although the reintroduction of infliximab treatment was safe, half of the patients did not achieve the same clinical response as prior to treatment withdrawal.

## Abbreviations

AS: Ankylosing spondylitis
ASAS: Assessment of Spondyloarthritis International Society
AxSpA: Axial spondyloarthritis
BASDAI: Bath Ankylosing Spondylitis Disease Activity Index
BASFI: Bath Ankylosing Spondylitis Functional Index
CRP: C-reactive protein
ESR: Erythrocyte sedimentation rate
IBD: Inflammatory bowel disease
NSAID: Nonsteroidal anti-inflammatory drugs
NY criteria: New York criteria
SD: Standard deviation
SER: Spanish Society of Rheumatology
VAS: Visual analogue scales
